# FOXP3^+^ Regulatory T Cells in Hepatic Fibrosis and Splenomegaly Caused by *Schistosoma japonicum*: The Spleen May Be a Major Source of Tregs in Subjects with Splenomegaly

**DOI:** 10.1371/journal.pntd.0004306

**Published:** 2016-01-05

**Authors:** Audrey Romano, Xunya Hou, Mathieu Sertorio, Hélia Dessein, Sandrine Cabantous, Pablo Oliveira, Jun Li, Sandrine Oyegue, Violaine Arnaud, Xinsong Luo, Martine Chavanieu, Odette Mariani, Xavier Sastre, Anne-Marie Dombey, Hongbin He, Yuesheng Li, Alain Dessein

**Affiliations:** 1 INSERM, UMR-906, Marseille, France; 2 Université Aix-Marseille, Faculté de Médecine, Marseille, France; 3 Hunan Institute of Parasitic Diseases, Yueyang, China; 4 INSERM U1040, Hôpital St Eloi, Montpellier, France; 5 Institut Curie, Section Médicale, Paris, France; 6 Etablissement Français du Sang, Marseille, France; 7 Assistance Publique, Hôpitaux de Marseille, Marseille, France; European Bioinformatics Institute, UNITED KINGDOM

## Abstract

*Schistosoma* eggs cause chronic liver inflammation and a complex disease characterized by hepatic fibrosis (HF) and splenomegaly (SplM). FOXP3^+^ Tregs could regulate inflammation, but it is unclear where these cells are produced and what roles they play in human schistosomiasis. We investigated blood and spleen FOXP3^+^ Tregs in Chinese fishermen with lifelong exposure to *Schistosoma japonicum* and various degrees of liver and spleen disease. FOXP3^+^ Tregs accounted for 4.3% of CD4^+^ T cells and 41.2% of FOXP3^+^CD4^+^ T cells; they could be divided into CD45RA^-^FOXP3^hi^ effector (eTregs) and CD45RA^+^FOXP3^low^ naive Tregs. Blood Treg levels were high in severe HF (+1.3; *p* = 0.004) and in SplM (+1.03, *p* = 0.03). Multivariate regression showed that severe HF (+0.85, *p* = 0.01) and SplM (+0.97; *p* = 0.05) were independently associated with the higher proportion of Tregs in the blood. This effect was mostly due to an increase in the proportion of eTregs in the blood of HF^+++^ (+0.9%; *p* = 0.04) and SplM (+0.9%; *p* = 0.04) patients. The proportion of eTregs expressing CXCR3 in the blood was lower in the HF^+++^ patients (37.4 +/- 5.9%) than in those with milder fibrosis (51.7 ± 2%; *p* = 0.009), whereas proportion were similar for cells expressing CD25^hi^, CCR7, and CTLA-4. Splenectomy improves symptoms and was associated with decreases in blood FOXP3^+^ Treg (-2.5; *p*<0.001) and eTreg (-1.3; *p* = 0.03) levels. SplM spleens contained a high proportion of eTregs with CXCR3, CCR5 and CTLA4 upregulation and CCR7 downregulation. This, and the strong expression of ligands of CXCR3 and CCR5 in the liver (*n* = 8) but not in the spleen suggested that spleen eTregs migrated to Th1-infiltrated liver tissues. Such migration may be attenuated in hepatosplenic patients due to lower levels of CXCR3 expression on Tregs (*p* = 0.009). Thus, higher blood Treg levels are associated with severe liver disease and splenomegaly. Our data are consistent with the hypothesis that the spleen is a major source of Tregs in subjects with splenomegaly. In most cases, Tregs migrate to the Th1-infiltrated liver and the lower levels of CXCR3^+^ Tregs in the blood of patients with severe schistosomiasis suggest that decreases in Treg migration sites of inflammation may aggravate the disease.

## Author Summary

Schistosomes are human parasites that cause severe hepatic disease in their host. They cause chronic inflammation when their eggs become trapped in small hepatic vessels. Most subjects from areas in which schistosomes are endemic display lifelong infection, and liver inflammation progresses to advanced hepatic fibrosis, portal hypertension and hypersplenism in 10 to 20% of infected subjects. The mechanisms controlling inflammation and limiting severe hepatic disease in most infected subjects remain unclear. We evaluated the role in this control of FOXP3^+^ Tregs, which exert strong control over inflammation. We found that activated FOXP3^+^ Treg levels were high in the blood of subjects with severe disease, probably due to the production of large numbers of these cells by the hyperactive spleen. We also found that the proportion of CXCR3^+^ effector FOXP3^+^ Tregs was lower, resulting in potentially lower migration rates and an aggravation of liver disease.

## Introduction

Regulatory T cells expressing the Forkhead box protein P3 (Foxp3) transcription factor are crucial regulators of immunological self-tolerance and homeostasis [1, 2]. They suppress the activation, proliferation and effector functions of many immune cells, including CD4^+^ and CD8^+^ T cells, natural killer cells, NKT cells, B cells, and antigen-presenting cells. The Treg phenotype results from two major regulatory events: the upregulation of genes associated with Treg function, including *FOXP3*, *CTLA4*, *IL2RA*, *TNFRSF18* (encoding GITR), *IKZF2* (encoding Helios) and *IKZF4* (encoding Eos), the expression of is epigenetically regulated [3, 4], and the FOXP3-mediated downregulation of several genes, including *IL2* and *IFNG* [5–8]. FOXP3^+^ Tregs have been divided into CD45RA^+^FOXP3^low^ CD4^+^ naïve Tregs and CD45RA^-^FOXP3^hi^CD4^+^ effector Tregs (eTregs), whereas blood CD45RA^-^FOXP3^low^ CD4^+^ T cells are effector T cells without suppressive activity [9, 10]. FOXP3^+^ Tregs are produced either in the thymus (tTregs), mostly by self-antigens, or in the periphery (pTregs) after stimulation by conventional antigens [11–13]. FOXP3^+^ Tregs regulate inflammation in response to infectious pathogens [14].

Schistosome worms lay their eggs in the mesenteric and portal veins of their human host; the eggs are trapped in liver sinusoids where they cause intense inflammation and fibrosis in the portal spaces. This, in turn, causes an increase in portal blood pressure and the development of varicose veins, leading to hemorrhage and death. In some patients, advanced hepatic fibrosis is associated with splenomegaly; this association is referred to as the hepatosplenic clinical form. Splenomegaly is invariably associated with a worsening of the disease, at least partly due to an aggravation of portal blood hypertension. However, the role of the spleen in severe schistosomiasis has been little explored and probably involves more than just a contribution to portal blood hypertension. We investigated the properties and fate of naïve and effector Tregs in the blood of *Schistosoma (S*.*) japonicum*-infected subjects with various degrees of hepatic fibrosis, with and without splenomegaly. The induction of pTregs should occur during egg-induced inflammation, but it may also occur in the hyperstimulated spleen of schistosome-infected individuals. Naïve Tregs express homing receptors for lymphoid organs (CCR7), whereas eTregs expressing high levels of CCR5, CXCR3, CCR6, and CCR8 [15] are attracted to non-lymphoid, inflamed tissues. Under these conditions, FOXP3^+^ Tregs become phenotypically and functionally specialized and develop into Th2, Th1 or Th17 cells [16]. It is unclear how mediators produced in the environment created by schistosome eggs influence Tregs. We first investigated the level of activation of FOXP3^+^ Tregs in the blood of *Schistosoma japonicum*-infected patients with schistosomiasis of various degrees of severity. We then evaluated homing receptors on Tregs and determined whether changes in Treg migration to the spleen and liver were associated with disease aggravation.

## Materials and Methods

### Ethics statement

The study was approved by the ethics committee of the Hunan Institute of Parasitic Diseases, Hunan Province, China and by the WHO. The French ethics committee did not authorize tests for HCV and HBV infection for the whole cohort. Only compliant participants were recruited and they were free to drop out at any point. Written informed consent was obtained from each subject.

### Evaluation of hepatic fibrosis

Hepatic fibrosis was evaluated by ultrasound and with the WHO grading scale [17], modified as described below. The WHO scale grades peripheral (NetF) and central fibrosis (CentF) separately. CentF is graded A, B, C, CL, D, E or F. The C linear thickening pattern (CL) of CentF represents the thickness of the uninterrupted fibrosis of the linear wall of the portal vein extending from the portal vein to its branches. The uninterrupted nature of the fibrosis distinguishes CL from grade C (discontinuous thickness), and the linear pattern differentiates it from the patches observed in grades D, E, and F. More than 60% of the fishermen had grade CL fibrosis. We therefore subdivided CL into CL^L^ (CL light), CL^M^ (CL medium) and CL^H^ (CL heavy): CL^L^ was observed in the left lobe of the liver only and CL^M^ and CL^H^ were observed in both lobes. Subjects with right lobe fibrosis extending to second-order branches were classified as CL^M^ and those with right lobe fibrosis extending well into the second-order branches were classified as CL^H^. Only CL^H^ was associated with evidence of portal hypertension, and was therefore grouped with grades D, E and F to define a severe CentF phenotype. The WHO grades peripheral fibrosis (network fibrosis, NetF) as narrow mesh (GN) when the lumen diameter of net was <12 mm across and wide mesh (GW) if >12 mm. We also refined GN grading into three categories: GN^L^ (or GW^L^) if the mesh streak (or band) was <2 mm thick, GN^M^ (or GW^M^) if 2–4 mm, and GN^H^ (or GW^H^) if >4 mm thick. Patients were assigned to three hepatic fibrosis (HF) groups on the basis of CentF: HF^+/-^ (B, C), HF^++^ (CL^L^) and HF^+++^ (CL^M^, CL^H^, D, E). Multivariate regression analysis showed that NetF had no effect on any of the dependent variables studied. We nevertheless indicate the NetF grade in our analysis: absent (G0) or light (GN^L^) in the HF^+/-^ group, and intermediate (GN^M^) or high (GN^H^, GW) in the HF^++^ and HF^+++^ groups. Study subjects were also assigned to two groups on the basis of spleen size: normal spleen (Spl, spleen size <110 mm) and splenomegaly (SplM, >110 mm). Patients who had undergone splenectomy were included in a separate Spl- group. Finally, individuals with moderate or severe hepatic fibrosis and splenomegaly were historically described as hepatosplenic patients.

### Study groups

All the subjects studied were fishermen working on the Dong Ting Lake who were recruited (from 2003 to 2009) from the same region, and were highly exposed to infection with *S*. *japonicum*. Exposure was evaluated by interviews, as previously described [18]. Only subjects with high levels of exposure were included in this study and exposure was not, therefore, a significant covariate in the analyses. In this population, we found no correlation between clinical disease and the number (0 to more than 20) of praziquantel treatments, probably because treatments were taken no more frequently than every two to three years, on average. Liver and spleen diseases were carefully evaluated by at least two ultrasound scans, carried out during different periods. It was not possible to perform such studies on a very large number of fishermen due to the long distance between the field and the laboratory.

### Group 1: Chinese fishermen for the FACS analysis of blood Tregs

The Chinese patients studied (*n* = 76) were from a large population of fishermen (a few thousand) investigated in a previous study [18]. They were selected according to the criteria mentioned above. All blood samples were collected and processed on the same day. All FACS analyses were performed within 36 hours of blood or tissue collection; none of the samples were frozen. Samples were from controls (*n* = 20) were collected and studied on the same days as those of the patients. The controls were living in the same region but reported no contact with lake water; they tested negative for schistosome antigens by ELISA and showed no signs of spleen or liver disease. All study subjects were aged between 30 and 65 years. Eleven of the 16 HF^+++^ patients, seven of the 23 HF^++^ patients and six of the 29 HF^+/-^ patients had splenomegaly and therefore also belonged to the SplM group. The splenectomy group (Spl-) included eight subjects with HF^++^ or HF^+++^.

### Group 2: Liver, blood and spleen tissues

All tissues were obtained from subjects undergoing splenectomy at Yueyang Hospital. These subjects came from the same population of fishermen (four men and four women) and they were 25–59 years old (48.2 ± 3.8). None of these individuals was infected with HCV or HBV and all had schistosome eggs in liver biopsy specimens. All but three had advanced or severe CentF or NetF. Three patients displayed milder but nevertheless significant CentF, which was associated with advanced NetF in two patients. These patients had severe splenomegaly. Control healthy tissues were obtained from a tissue bank in France. Liver biopsy specimens were collected from eight patients; blood and spleen tissues were obtained from five (four men and one woman) of these eight subjects.

### Antibodies and flow cytometry

All cell labeling was performed on cells immediately after their purification from the blood, without stimulation. Counts and viability were determined with a hemocytometer and the trypan blue dye exclusion technique. An average of 95% of the cells were viable cells. PBMCs or spleen cells were dispensed (4 x 10^5^ cells/tube) into 5 ml polystyrene tubes (Falcon) for surface and intracellular staining with the Human FOXP3 Buffer Set (BD Pharmingen). Quadruple staining was carried out with FITC-conjugated anti-CD45RA (H1100; BD Pharmingen), PE-Cy7-conjugated anti-CD4 (L3T4, eBiosciences), and PE-conjugated anti-FOXP3 (259D/C7; BD Pharmingen) antibodies, together with one of the following APC-conjugated antibodies: anti-CD25 (M-A251; BD Pharmingen) antibodies; anti-CCR7 (3D12; eBiosciences), anti-CXCR3 (1C6/CXCR3; BD Pharmingen), anti-CCR5 (2D7/CCR5; BD Pharmingen) or anti-CTLA-4 (BNI3; BD Pharmingen) antibody. For the analysis of IFN-γ production, cells were first double-stained with FITC-conjugated anti-CD3 (UCHT1; BD Pharmingen) and PE-Cy7–conjugated anti-CD4 antibodies and then stained with APC-conjugated anti-IFNγ intracellular markers (4S-B3; eBiosciences). They were incubated with 100 ng/ml PMA, 1 μg/ml ionomycin and monensin (BD GolgiStop) for 6 hours at 37°C before intracellular cytokine labeling. Isotype controls were obtained from the corresponding manufacturers. All antibodies were used according to the manufacturers’ recommendations. Flow cytometry was carried out with a FACScalibur flow cytometer (BD Biosciences) and cellquest software. DIVA and FlowJo software (TreeStar) software was used for analysis.

### RNA extraction and quantitative RT-PCR

Liver and spleen biopsy specimens were stored in RNA Later (Life Technologies, Courtaboeuf, France) at -20°C. Tissue homogenization was carried out with a Precellys-24 device (Bertin Technologies, Ozyme, Saint-Quentin-en-Yvelines, France), with ceramic beads (1.4 mm diameter, CK14), in 350 μl RLT lysis buffer (Qiagen SAS, Courtaboeuf, France) supplemented with 3.5 μl β-mercaptoethanol. We added 400 μl of Tri-reagent (Life Technologies) and 150 μl of chloroform. The aqueous phase was mixed with 500 μl of 50% ethanol (liver) or 70% ethanol (spleen), and RNA was purified on an RNeasy spin column (Qiagen SAS, Courtaboeuf, France). RNA integrity was assessed with a 2100 Bioanalyzer (Agilent, Palo Alto, CA, USA). Liver and spleen “controls” were from the Biological Resource Center, Curie Institute, Paris, and from Stratagene (Agilent), Clonetech (Ozyme), Panomics (Ozyme), and INSERM U1040, Montpellier. Biopsy specimens were collected from deceased individuals with no known history of infection (i.e. from untransplanted organs) or from liver biopsies carried out for diagnostic purposes. We checked that the donors were healthy, by assessing inflammatory cytokine levels in these tissues. If a “healthy tissue” displayed an abnormal pattern of inflammatory cytokine expression (with respect to that in the other biopsies), it was excluded from the study. Total RNA (1 μg), RIN > 7, was reverse-transcribed with the High Capacity cDNA Reverse Transcription Kit (Life Technologies, Courtaboeuf, France). Real-time quantitative PCR, with 20 ng of cDNA, was performed with the ABI 7900HT Fast Real-Time PCR System and *Taq*Man Universal PCR Master Mix (Applied Biosystems, Life Technologies). The *Taq*Man gene expression assays used in this study were as follows: *CCL3* (Hs00234142_m1), *CCL5* (hs00174575_m1), *CCL19* (Hs00171149_m1), *CCL20* (Hs00171125_m1), *CCL21* (Hs99999110_m1), *CXCL9* (Hs00171065_m1), *CXCL10* (Hs00171042_m1), *CXCL11* (Hs00171138_m1), *IFNG* (Hs99999041_m1), *IL12B* (Hs00233688_m1), *IL12RB2* (Hs00155486_m1), *RPLP0* (Hs99999902_m1), *TBX21* (Hs00203436_m1), from Applied Biosystems. Gene expression values were normalized relative to those for the housekeeping gene *RPLP0* (ribosomal phosphoprotein large P0). Transcript levels for this housekeeping gene were stable in all the study groups. A significant difference (a value differing from the mean for the other samples by more than twice the SEM) in the abundance of *RPLP0* transcripts between one sample and the mean value for the other samples was considered to indicate a problem with RNA extraction.

### Statistical analysis

Group comparisons were performed by nonparametric analysis in SPSS software. We assessed how hepatic disease (fibrosis), spleen disease (splenomegaly) and splenectomy affected subpopulations of Tregs, by carrying out linear regression analysis on these dependent variables. Hepatic fibrosis was divided into three binary classes, as previously described [19]. The variables introduced into the regression model were made binary to avoid making assumptions about the existence of a linear relationship between the dependent variable and the independent variables. Spleen disease was also divided into three binary classes. All binary variables were included in the linear regression model. Age and sex were not significant covariates in most models. Results are given as the non-standardized slope (A), its 95% confidence interval (CI) and the *p* value of the association. The statistical significance of the effect of splenectomy was systematically assessed by comparisons with the splenomegaly group or the HF^+++^ group, because all the splenectomized subjects belonged to these groups before surgery. Praziquantel treatment varied considerably between fishermen and was included as a covariate. Surprisingly, despite considerable effort, we found no correlation between the number of praziquantel treatments (0 to >20) and disease intensity or Treg response. Exposure was not included as a covariate because all the study subjects had high levels of exposure to the infected waters of the lake. As independent testing was carried out, *p* values above 0.01 are suggestive of an association and the corresponding variables will be investigated again in a future study on a different population. No correction method was used because multivariate analyses involving nested models do not require statistical correction.

## Results

### Effector and naïve Tregs in the blood of individuals infected with *S*. *japonicum*

**Fig 1:**
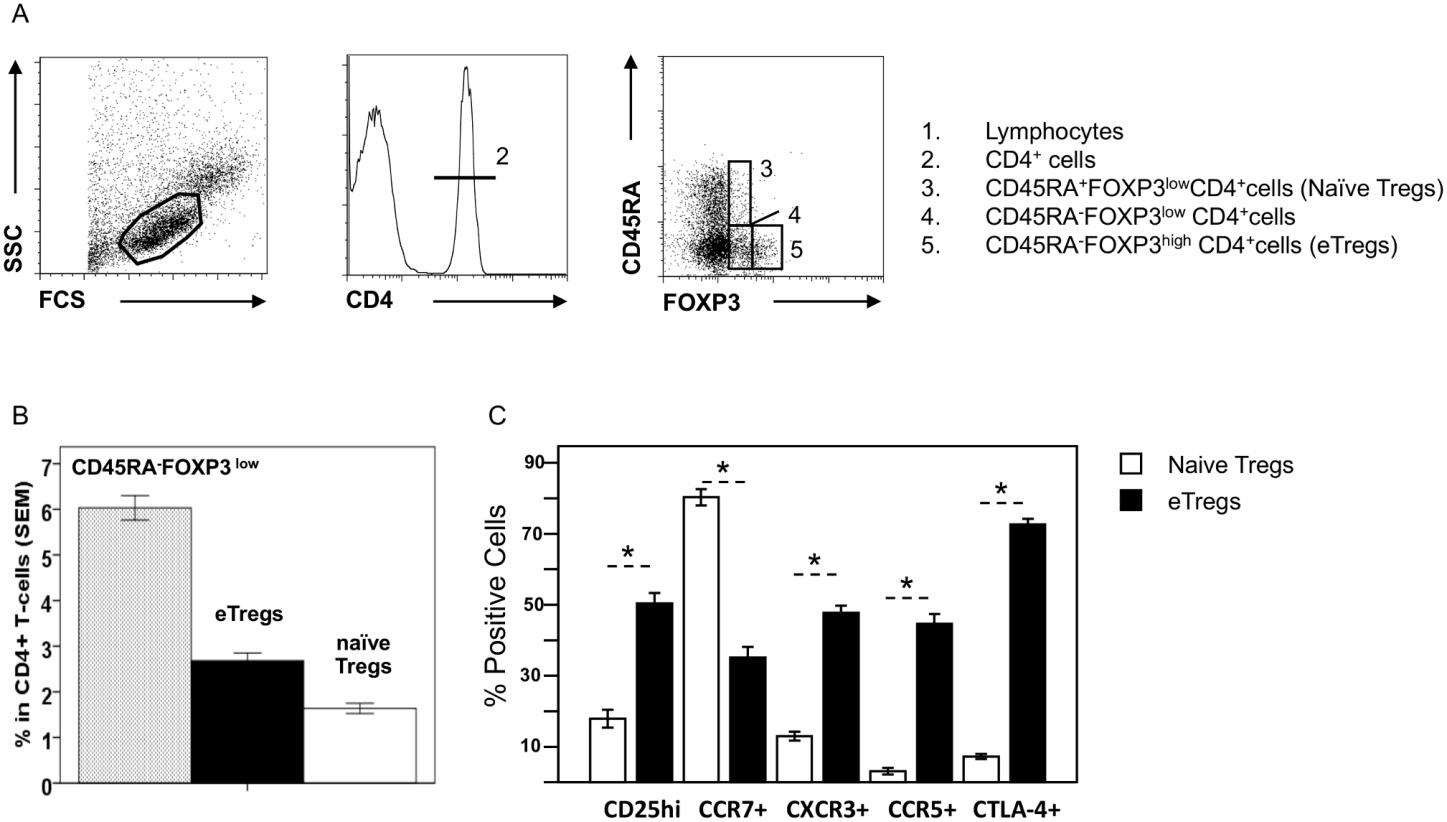
Phenotyping of FOXP3^+^ Tregs. **A**) Gating strategy for accurate determination of the proportion of regulatory T cells in PBMCs 1. Lymphocytes; 2. CD4^+^ T cells; 3. CD4^+^CD45RA^+^FOXP3^low^ T cells; 4. CD4^+^CD45RA^-^FOXP3^low^ T cells; 5.CD4^+^CD45RA^-^FOXP3^high^ T cells); **B**) Percentage of blood CD45RA^-^FOXP3^low^ non-Tregs (gray bars), CD45RA^-^FOXP3^high^ T cells (black bars) and CD45RA^+^FOXP3^low^ T cells (white bars) among the blood CD4^+^ T cells of infected patients (group 1; *n* = 76); **C**) Proportion of CD45RA^-^FOXP3^high^ T cells and CD45RA^+^FOXP3^low^ T cells expressing CD25, CCR7, CXCR3, CCR5, or CTLA-4 among blood CD4^+^ T cells in 45 patients (excluding splenectomized patients). A nonparametric test was used. * *p*<0.01.

We evaluated the proportion of Tregs among blood FOXP3^+^ T cells in all the fishermen (group 1). These individuals had been exposed to the risk of infection with *S*. *japonicum* for more than 10 years. Tregs (CD45RA^+^FOXP3^low^ and CD45RA^-^FOXP3^hi^) accounted for 4.3 ± 0.26% (SEM) of all CD4^+^ T cells and 41.2 ± 0.16% of all FOXP3^+^CD4^+^ T cells (Fig 1A and 1B). The remaining FOXP3^+^CD4^+^ cells, CD45RA^-^FOXP3^low^ T cells, corresponding to FOXP3^+^ non-regulatory T cells, accounted for 6.1 ± 0.4% of all CD4^+^ T cells and 58.7 ± 0.15% of the FOXP3^+^CD4^+^ T cells.

We phenotyped patient CD45RA^-^FOXP3^hi^ and CD45RA^+^FOXP3^low^ Tregs for CD25, CCR7, CXCR3, CCR5 and CTLA-4 (splenectomized patients were excluded from this analysis) (Figs 1C and S1). CCR7 directs Tregs to lymphoid organs and CXCR3 and CCR5 direct these cells to Th1-infiltrated tissues [20]. Strong CD25 expression is a marker of Treg activation, whereas CTLA-4 expression is strongly correlated with suppressive activity. Most CD45RA^+^FOXP3^low^ Tregs expressed CCR7 (80.5 ± 1.2%), but a few expressed CD25 (18.3 ± 2.3%), CXCR3 (11.6 ± 2%), CCR5 (3.2 ± 0.8%) and CTLA-4 (8.3 ± 0.7%). By contrast, the proportion of cells expressing CCR7 (34.1 ± 2.2%) was lower in CD45RA^-^Foxp3^hi^ than in CD45RA^+^Foxp3^low^ Tregs (*p*<0.01). By contrast the proportions of cells with CD25^hi^ (51.2 ± 2.2%), CXCR3 (48.1 ± 2%), CCR5 (45.8 ± 2.1%) or CTLA-4 (71.7 ± 1.5%) expression were higher for CD45RA^-^FOXP3^hi^ than for CD45RA^+^FOXP3^low^ Tregs (*p* <0.01 for all comparisons). These patterns were used to characterize CD45RA^+^FOXP3^low^ naive Tregs and CD45RA^-^FOXP3^hi^ eTregs, respectively in normal blood [9]. Thus, blood Tregs from schistosome-infected patients can be divided into eTregs and naïve Tregs.

### Both severe fibrosis and splenomegaly are independently associated with an increase in blood Treg levels

Splenomegaly (SplM) occurs in patients with advanced hepatic fibrosis (HF). Tregs may be produced and/or attracted to both the liver and spleen, because these organs are sites of intense inflammation and cell proliferation. We evaluated the frequency of Tregs in the blood of individuals (study group 1) with spleen and/or liver disease of various degrees of severity, to investigate a possible link between SplM, HF and Tregs. Study subjects were assigned to three groups according to the severity of HF and to two groups on the basis of spleen size, as described in the methods.

**Fig 2:**
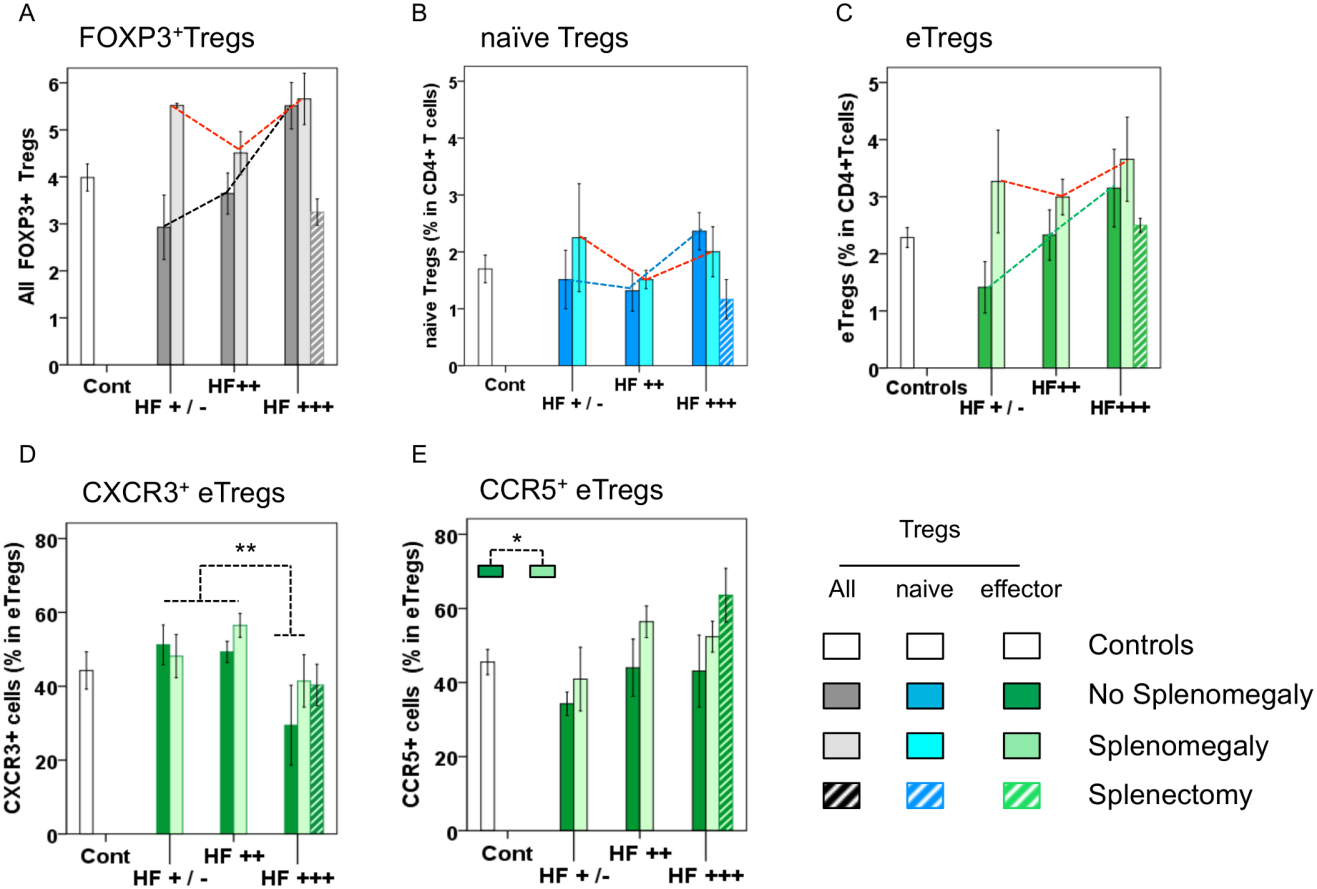
Proportion of FOXP3^+^ Tregs according to patient clinical status. **(A**) Proportions of total FOXP3^+^ Tregs (gray bars); **B**) naïve Tregs (blue bars); **C**) and eTregs (green bars) in the blood of study subjects, by clinical status (hepatic fibrosis grade and splenomegaly); **D**) Proportion of CXCR3^+^ cells among eTregs, by clinical status, **E**) Proportion of CCR5^+^ cells among eTregs, by clinical status. Fig 2A: Blood FOXP3^+^ Treg levels were positively correlated with the severity of both HF (*p* = 0.004) and SplM (*p* = 0.03). In the multivariate regression analysis, HF (*p* = 0.01) and SplM (*p* = 0.05) were associated with high blood Treg levels. Fig 2C: Blood eTreg levels were higher in patients with HF (*p* = 0.05) or SplM (*p* = 0.08) than in healthy controls. The statistical differences shown in Fig 2E correspond to the comparison of HF^+/-^ and HF^++^ with HF^+++^ (51.7 ± 2%; *p* = 0.009). Subjects with splenomegaly were compared with subjects with a normal spleen (*p* = 0.04). All patients are from group 1 (Cont *n* = 20, HF^+/-^ *n* = 29; HF^++^ *n* = 23; HF^+++^ *n* = 16, normal spleen *n* = 44, splenomegaly patients *n* = 24, splenectomized subjects *n* = 8). * *p*<0.05; ** *p*<0.01.

We found that the levels of all Tregs and of eTregs in the blood increased with increasing hepatic fibrosis grade (Fig 2A (all Tregs) and Fig 2C (effector Tregs); black and green dotted lines). However, this effect was not observed in subjects with splenomegaly (dotted red lines), because splenomegaly markedly increases blood Treg and eTreg levels regardless of the degree of hepatic fibrosis (for all HF grades: HF^+/-^, HF^++^ HF^+++^). However, we observed no clear effect of HF (dotted blue line) and splenomegaly (dotted red line) on blood levels of naïve Tregs (Fig 2B). Thus, our findings suggest that both hepatic fibrosis and splenomegaly increase the levels of all Tregs and of eTregs in the blood. This was confirmed by the simultaneous testing of HF and SplM in the regression model, which showed that HF (+0.85 ± 0.3%, *p* = 0.01) and SplM (+0.97 ± 0.49%, *p* = 0.05) were independently associated with high blood Treg levels. Furthermore, eTregs and naïve Tregs were not equally affected. Blood eTreg levels (Fig 2C) were higher both in patients with HF (*p* = 0.05) and in patients with SplM (*p* = 0.08) than in healthy controls, but the levels of naïve Tregs in the blood were similar in these two groups (Fig 2B). The largest differences observed were those between eTreg levels in HF^++^ and HF^+++^ patients (+0.9 ± 0.4%, *p* = 0.04) and between SplM patient and patients with a normal spleen (+0.9 ± 0.36%, *p* = 0.04).

### The proportion of CXCR3^+^ eTregs in the blood is lower in patients with severe hepatic fibrosis, and the proportion of CCR5^+^ eTregs may be higher in patients with splenomegaly (study group 1)

We analyzed the expression of molecules crucial for the activation and homing of Tregs, to evaluate the migratory capacities of these cells. We also evaluated CTLA-4, a marker of suppressor activity. The proportions of eTregs expressing CD25^hi^, CCR7, and CTLA-4 were similar between the Spl and SplM group and among HF groups. However, the proportion of CXCR3^+^ eTregs was lower in the HF^+++^ group (37.4 ± 5.9%) than in patients with milder fibrosis (HF^+/-^ and HF^++^, as compared to HF^+++^) (51.7 ± 2%; *p* = 0.009) and was not affected by SplM (Fig 2D). There seemed to be a higher proportion of CCR5^+^ eTregs (Fig 2E) in subjects with splenomegaly (*p* = 0.04) than in subjects with a normal spleen, regardless of the degree of hepatic fibrosis. There are, thus significantly fewer CXCR3^+^eTregs in the blood of hepatosplenic subjects than in the blood of subjects with milder disease. Conversely, our data suggest that the proportion of CCR5^+^ eTregs may be higher in patients with splenomegaly, but it proportion may not be affected by the degree of hepatic fibrosis.

### The genes encoding IFN-γ-dependent chemokines, ligands for CXCR3 and CCR5, are highly transcribed in the liver of HSP subjects (study group 2)

**Fig 3:**
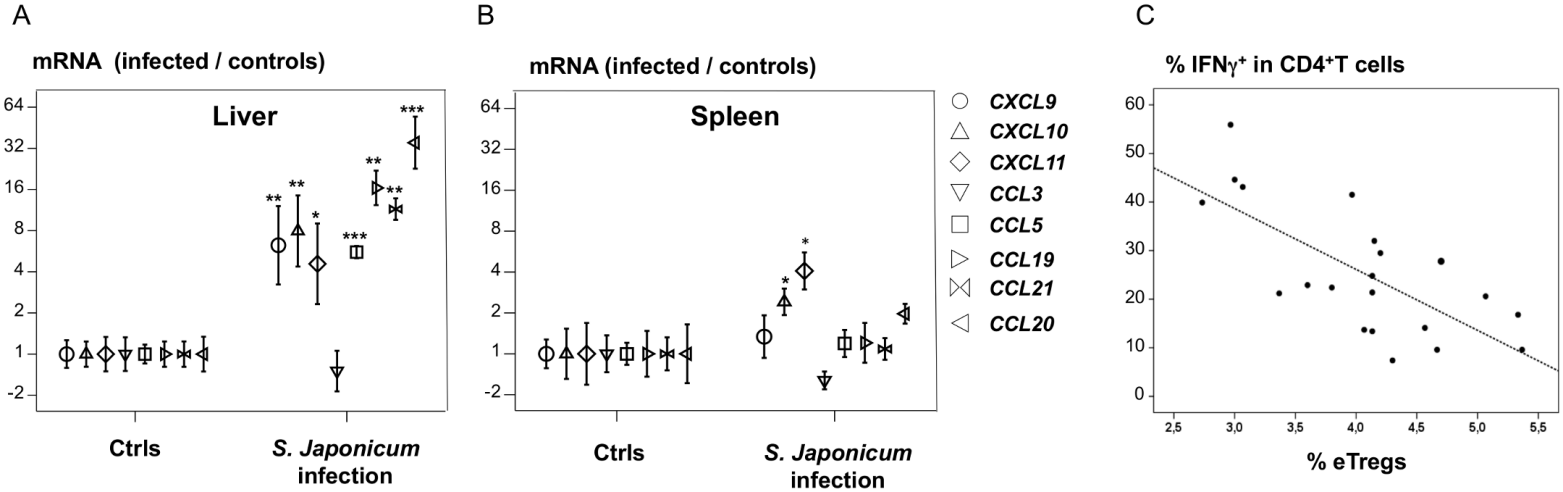
Ligands for CXCR3 and CCR5 are overexpressed in the livers of hepatosplenic subjects. The highest proportions of Tregs among PBMCs were associated with the lowest levels of IFNγ production by PBMCs. A-B) Expression of the ligands for *CXCR3*, *CCR5* and *CCR7* in the liver and spleen of eight hepatosplenic patients (Group 2). Messenger RNA levels are expressed relative to the arithmetic mean values obtained for 11 healthy controls. **C**) The proportion of IFNγ^+^ cells among blood CD4^+^ T cells is negatively correlated (*r* = -0.73, *p* = 0.002) with the proportion of eTregs in the blood in Group 1. The proportion of IFNγ^+^CD4^+^ T cells was determined after 6h of stimulation with PMA, ionomycin and monensin. Nonparametric statistical tests were used * *p*<0.01, ** *p*<0.001, *** *p*<0.0001.

CXCR3 and CCR5 direct FOXP3^+^ Tregs to sites infiltrated with T_H_1 cells. We therefore hypothesized that CXCR3 and CCR5 regulated the trafficking of eTregs toward Th1-infiltrated egg granulomas in the liver. We tested this hypothesis by evaluating the production of CCR5 and CXCR3 ligands in liver biopsy specimens from hepatosplenic patients who underwent splenectomy (study group 2). We also evaluated the production of CCR7 and CCR6 ligands, because these receptors are also expressed by FOXP3^+^ Tregs (this study and [15]). Transcript levels for *CCL5* (CCR5 ligand), *CXCL9*, *10*, *11* (CXCR3), *CCL19*, *CCL21* (CCR7) and *CCL20* (CCR6) were four to 33 times higher (p<0.01) in infected than in control livers (Fig 3A). The largest difference in expression detected was that for *CCL20* (33-fold, *p*<0.001). By contrast, in the spleen, only *CXCL11* (CXCR3) and *CXCL10* (CXCR3) transcripts were more abundant in HSP patients than in control individuals (*p*<0.01) (Fig 3B).

We also evaluated transcripts of T_H_1-related genes in infected livers: *IL12RB2* mRNA levels (*p* = 0.004) were four to five times higher in infected than in control livers, and *IFNγ*, *IL12B* and *TBX21* mRNA levels followed a similar pattern (*p*<0.15). Moreover, transcripts of *CXCR3*, *CXCL9* (*r* = 0.93, *p* = 0.02) and *CXCL11* (*r* = 0.88, *p* = 0.05) were correlated with *IFNG* transcript levels in the liver, but not in the spleen of infected subjects (*CXCL10* also showed a trend towards correlation; *r* = 0.79 *p* = 0.1). No such correlation was found for the other chemokines tested: *CCL3* (ligand for *CCR1*, *5*), *CCL5* (*CCR1*, *3*, *5*), *CCL19* (*CCR7*), *CCL20* (*CCR6*), *CCL21* (*CCR7*) and *CXCL9* (*CXCR3*). Similarly, eTreg levels in the blood were negatively correlated (*r* = -0.73, *p* = 0.002) with T_H_1 cell levels in the blood (Fig 3C), consistent with the negative regulation of T_H_1 cells exerted by e Tregs.

### The spleens of patients with severe disease (requiring splenectomy) contain high proportions of effector Tregs (study group 2)

**Fig 4:**
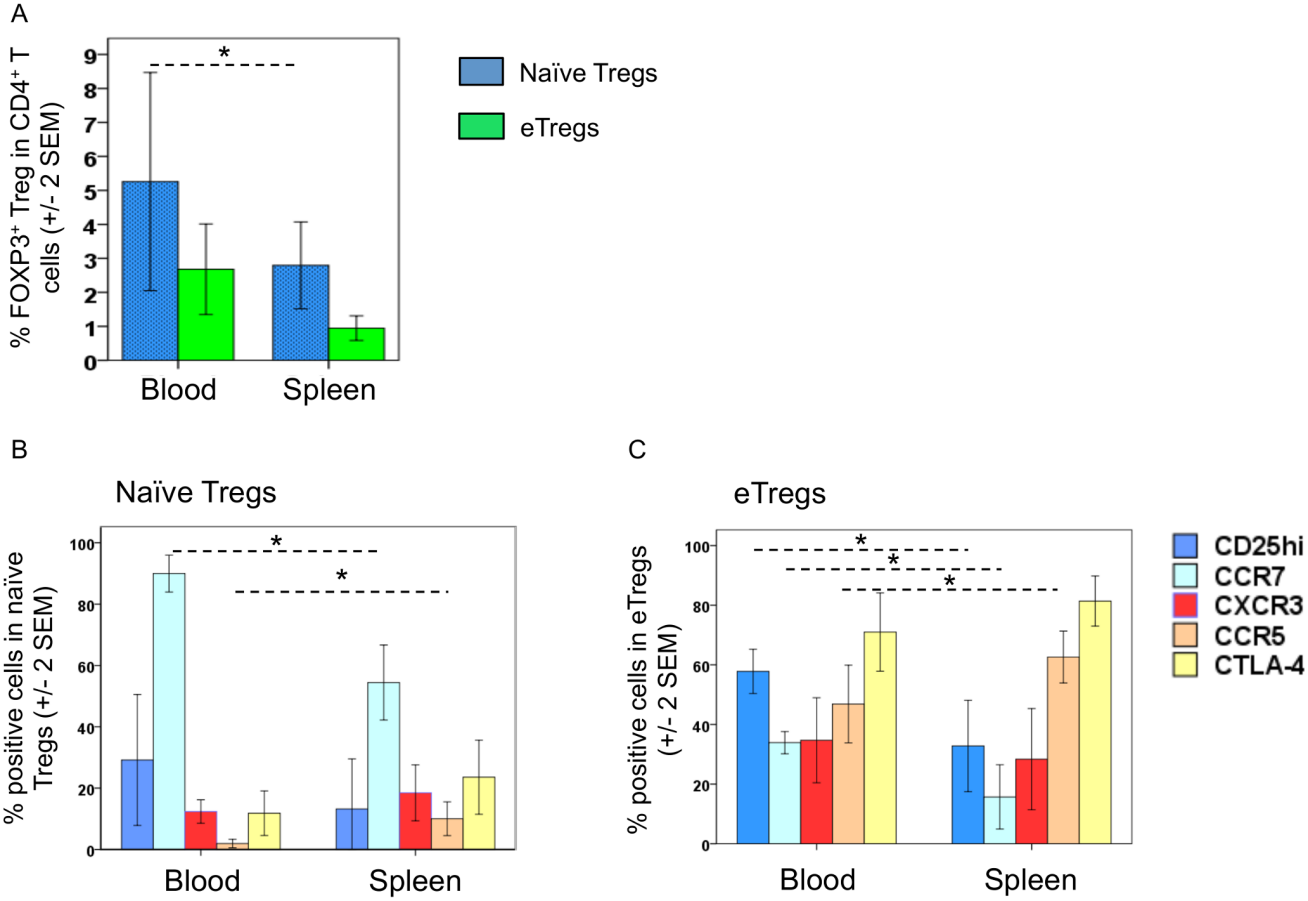
Proportions of naïve (blue bars) and effector (green bars) Tregs in the blood and spleen of subjects with severe spleen and liver disease (hepatosplenic subjects) requiring splenectomy. (A) Proportions of eTregs (B) and naïve Tregs (C) expressing CD25hi, CCR7, CXCR3, CCR5 and CTLA-4 in the blood and spleen of these same hepatosplenic subjects. Data from five patients with severe disease are shown (Group 2). Nonparametric tests were used *p<0.05.

We found that blood levels of FOXP3^+^ Tregs (-2.5 ± 0.56, *p*<0.001) and eTregs (-1.3±0.6, *p* = 0.03) were lower in patients with severe liver (HF^++^ or HF^+++^) and spleen (SplM) disease who had undergone splenectomy (Fig 2A and 2C) than in HF^+++^ subjects with splenomegaly. This observation, in addition to the high Treg counts in the blood of patients with SplM, suggests that the spleen may be involved in eTreg production (induction or proliferation). We thus analyzed Tregs in the spleens removed from patients with severe hepatosplenic disease (Fig 4). The proportion of naïve Tregs among CD4^+^ T cells was lower in the spleen (0.95 ± 0.2) than in the blood (2.68 ± 0.7) (*p* = 0.02) (Fig 4A). The proportion of naïve Tregs expressing CCR7 was also lower in the spleen than in the blood (*p* = 0.006) (Fig 4B). However, the proportion of naïve Tregs expressing CCR5 tended to be higher in the spleen than in the blood (*p* = 0.07) (Fig 4B). There was no statistically significant difference in the proportion of eTregs in the spleen (2.8 ± 0.64) and blood (5.2 ± 1.6) (Fig 4A). However, the proportion of eTregs expressing CD25 or CCR7 was lower in the spleen than in the blood (*p* = 0.05 and *p* = 0.04, respectively) whereas the proportion of eTregs expressing CCR5 tended to be higher in the spleen than in the blood (*p* = 0.08) (Fig 4C). Thus the composition of the naïve Treg and eTreg populations differed between the spleen and the blood. Spleen eTregs may be less activated than blood eTregs. Nevertheless, spleen eTregs displayed higher levels of CCR5 and CTLA-4 expression, typical of cells committed to migrate to inflamed tissues. The observation that spleen naïve Tregs have weaker CCR7 expression and stronger CCR5 expression than blood naïve Tregs suggests that they have been activated, a process that might ultimately lead to their transformation into eTregs.

## Discussion

Few studies have analyzed FOXP3^+^ Tregs in patients infected with schistosomes and no other study has focused on FOXP3^+^ Tregs without the interference of FOXP3^+^ non-Tregs. Indeed, to define human Treg cells, many studies used the following combination of markers CD4^+^CD25^hi^FOXP3^+^ or CD4^+^CD25^hi^FOXP3^+^CD127^-/low^, however they don’t allow the exclusion of FOXP3^+^ non-Tregs cells [21]. Therefore, being limited by a four colors cytometer, we chose to use the CD45RA marker to eliminate the FOXP3^+^ non-Tregs cells from the study as well as analyzing the naïve versus effector Tregs populations. Although others have described that naïve Tregs and eTregs are CD127^-/^low, this marker combination might underestimate the frequencies of Tregs [9, 21], therefore, it will be necessary in future study, to analyze simultaneously additional markers such as CD127, Helios, Ki67 to better define the Treg cells populations.

Thus, we studied naïve Tregs and effector Tregs separately, which relate to different stages of differentiation/activation of the Treg population. Effector Tregs are partly derived from the activation of naïve Tregs. They may also be generated by eTreg multiplication. Peripheral Tregs (pTregs) and thymic Tregs (tTregs) are named according to the part of the body in which Treg differentiation occurred, either during thymic development or after birth. Tregs can be induced (induced Tregs) from pTregs and tTregs and it is generally accepted that inducible Tregs are produced in peripheral organs and require various stimuli that are probably best delivered in a lymphoid environment. Most of the reported observations relate to mice. In humans, the origin and fate of inducible Tregs are less clear. In this work, the modulation of blood Treg and eTreg levels and our observations for spleen Tregs are indicative of Treg induction in the periphery by signals such as egg-derived molecules. Our data are also consistent with the occurrence of induction in the spleen of hepatosplenic subjects.

We found that both fibrosis and spleen disease were independently associated with high FOXP3^+^ eTreg levels in the blood. Others have shown that CD4^+^CD25^+^FOXP3^+^CD127^low^ T-cell levels are high in the blood of *S*. *haematobium*-infected children and in the blood of *S*. *mansoni*-infected individuals after anti-helminthic treatment [22, 23]. These results, together with those presented here, indicate that schistosome infection stimulates the production of FOXP3^+^ Tregs. The positive correlation between eTreg frequencies in the blood and the severity of both hepatic and spleen disease suggests that infection stimulates the production or regulates the induction of Tregs. Such induction may occur in lymphoid organs strongly simulated with schistosome eggs, such as the spleen or the mesenteric lymph nodes. Our findings suggest that Treg induction may occur in the spleen of subjects with splenomegaly. We found that the spleens of these patients contained a large proportion of Tregs, most of which were already activated, although not to the same extent as the Tregs in the blood. Thus, the spleen may be a source of Tregs, particularly given its large size in patients with splenomegaly. Alternatively, spleen Tregs may be produced in the blood and captured by the spleen. However, this seems unlikely because splenomegaly in patients was associated with the highest eTreg levels in blood and splenectomy resulted in a drop in blood eTreg levels. Moreover, the properties (high proportions of CXCR3, CCR5 and CTLA-4) of spleen eTregs indicated that these cells were unlikely to remain in the spleen, instead being poised to migrate to inflamed tissues, such as tissues infiltrated with large numbers of eggs, such as the liver and the intestine. Unlike the spleen, the liver displayed high levels of CXCR3 and CCR5 ligands and Th1 inflammation known to attract CXCR3^+^ and CCR5^+^ Tregs. The blood and spleen Tregs may therefore home to the liver rather than remaining in the spleen. However, we cannot rule out the possibility that unknown mechanisms prevent Treg egression from the spleen in patients with splenomegaly, causing Treg accumulation in this organ. We studied RNA levels in the liver with non-endemic controls (control tissues from a French blood bank) because we could not obtain local controls (there was no local tissue bank). The differences reported here may not be entirely due to schistosome infections, instead reflecting genetic differences between controls and patients. We recently performed transcriptome analyses on these samples and found that only 50 genes displayed increases at least as important as those reported here for the chemokine receptor ligands. We therefore think it highly unlikely that the differences observed were not specific to the infection. Thus, our data suggest that some of the blood eTregs in schistosome-infected patients are produced in the spleen (pTregs), either by induction from naïve Tregs or by the proliferation of eTregs. The induction of peripheral FOXP3^+^ Tregs has been demonstrated in mice [11–13]. It is triggered mostly by conventional antigens and results in the selection of high-affinity TCRs. These cells have been less studied in human diseases, due to the lack of markers for distinguishing human pTregs from tTregs. However, in a recent report, neuropilin was identified as a marker of tTregs [24, 25] in mice. If this result is subsequently confirmed in humans, then it will be possible to use neuropilin and proliferation markers, such as Ki67, to distinguish between tTregs and pTregs and to demonstrate definitively the active production of eTregs in the spleen and, possibly, in other tissues hyperstimulated by eggs, such as the mesenteric lymph nodes, through induction, proliferation, or both.

Our findings also suggest a possible explanation for high eTreg levels in the blood of both HF and SplM patients. First, a low proportion of CXCR3^+^ eTregs may limit the recruitment of eTregs to the liver. Second, the spleen of hepatosplenic patients may release larger numbers of Tregs into the blood, and, finally, the liver and mesenteric nodes may contribute to Treg production, as suggested in patients infected with HCV [26]. It is important to determine the role of Tregs in human schistosomiasis, because splenectomy, which is performed in hepatosplenic patients, may eliminate a major source of Tregs. The elimination of CD25^+^ T cells (accounting for >50% of non-Tregs) promotes collagen deposition in the liver of *S*. *mansoni*-infected mice [27]. However, the same treatment reduces worm and egg load in *S*. *japonicum*-infected animals, suggesting that Tregs may decrease the clinical manifestations of schistosomiasis but prevent the development of sterile immunity [28, 29]. However, these results require confirmation and no study has yet assessed the effects of highly enriched preparations of FOXP3^+^ Tregs in schistosome-infected mice.

Our finding that the proportion of CXCR3^+^ eTregs is lower in patients with severe HF indirectly supports the hypothesis that impaired Treg recruitment in the liver contributes to disease, although we cannot exclude the possibility that CXCR3^+^ Tregs are retained selectively in other organs, such as the spleen. Nevertheless, this conclusion is consistent with several reports showing that CXCR3 is crucial for the localization of Tregs in the inflamed liver [30, 31]. Helbig *et al*. [32] reported that the CXCR3 chemokines were the most strongly expressed chemokines in the livers of patients with chronic hepatitis C. Others have more directly implicated Foxp3^+^ Tregs in protection against chronic hepatitis: in a model of autoimmune inflammation of the liver, Lapierre *et al*. [33] observed that the adoptive transfer of *ex vivo*-expanded CXCR3^+^ Tregs in mice with auto-immune hepatitis deficiency resulted in targeting to the inflamed liver and the restoration of peripheral tolerance, inducing a remission of auto-immune disease. Furthermore Hasegawa *et al*. [34] showed that acute GVHD could be improved in the intestine, liver and lungs by the accumulation of CXCR3-expressing CD4^+^CD25^+^ regulatory T cells (but not CXCR3-Tregs) in target organs. CXCR3^+^ Treg cells accumulated in Th1-associated chemokine-expressing target organs, resulting in a stronger suppression of alloreactive donor T cells. Interestingly, Oo *et al*. [35] compared blood- and liver-derived Tregs and showed that liver-derived Tregs expressed large numbers of CXCR3 chemokine receptors. In flow-based adhesion assays with human hepatic sinusoidal endothelium, Tregs used CXCR3 for binding and transmigration. The authors suggested that CXCR3 mediated the recruitment of Tregs via the hepatic sinusoidal endothelium. Erhardt *et al*. [36] reported that CXCR3^+^Foxp3^+^ Tregs generated in mice with ConA–induced hepatitis disseminated in the body and migrated specifically to the liver, where they limited immune system-mediated liver damage. Finally, mice lacking CXCR3 are more prone to liver fibrosis initiated by the loss of the anti-fibrogenic and angiostatic effects of CXCL9 on hepatic stellate cells [37] and sinusoidal endothelial cells [38]. These and our findings suggest that the small proportion of CXCR3^+^ eTregs in individuals with severe schistosomiasis may impair the influx of eTregs into the liver, thereby contributing to HF. No such association with disease was observed with CCR5^+^ eTregs, the proportion of which in the blood may be increased in subjects with splenomegaly, as suggested here. If confirmed in a larger number of subjects, these results would favor attempts to compensate for the decrease the frequencies of CXCR3^+^ eTregs. It would be interesting to investigate the respective roles of these receptors in the migration to the different egg-infiltrated tissues, including the intestine, where they may also play important regulatory roles.

The role of FOXP3^+^ Tregs in the spleen, which does not contain schistosome eggs, is less clear. Tregs may limit inflammation in the spleen, thereby inefficiently containing the splenomegaly. Conversely, our observations raises the intriguing possibility that eTregs generated in the spleen of hepatosplenic patients may be pathogenic. Tregs are normally stable due to both TSDR demethylation and the FOXP3-mediated suppression of IL2. However, IL-2 activation may cause these cells to lose FOXP3 expression and their suppressive capacities [8]. However, in normal physiological conditions, they conserve their TSDR demethylation pattern, and this prevents them from becoming pathogenic. However, Tregs produced in the massive hyperplasic spleen of hepatosplenic patients may not acquire the epigenetic demethylation pattern of normal Tregs, like the Tregs of lymphopenic mice, which develop pathogenic properties [39]. Thus, Tregs generated in a huge, hyperactive spleen may be unstable and develop into pathogenic T cells (e.g. Th17 cells) aggravating spleen disease. We are currently evaluating this possibility.

The observation that splenomegaly is associated with high counts of potentially unstable Tregs that may develop pathogenic properties may also stimulate research into Tregs in malaria and visceral leishmaniasis, which are also associated with marked splenomegaly. In both infections, the liver and the spleen play important roles in controlling parasite multiplication. It is therefore essential to confirm that the spleens of individuals with splenomegaly overproduce eTregs and to check whether these eTregs present the epigenetic signature of stable suppressive Tregs.

In summary, blood and spleen Treg levels are increased in individuals with severe hepatosplenic disease caused by *S*. *japonicum*. An analysis of the homing receptors on these cells and of the receptor ligands in the liver suggest that these cells may migrate to the liver (and probably also the intestine), to contain Th1 inflammation. Treg migration to tissues may be reduced by impaired CXCR3 expression on these cells. Further investigations are required to confirm these observations in a larger number of individuals with different clinical forms of schistosomiasis as well as the inclusion in the study of additional markers to define Tregs populations.

## Supporting Information

S1 Fig Phenotyping of naïve Tregs and eTregs. A) Representative dotplos showing isotypes control antibodies staining for FOXP3 and CD45RA B) Representative dotplots of the proportions of CD25^hi^, CCR7^+^, CXCR3^+^, CCR5^+^ and CTLA-4^+^ cells among naïve Tregs and eTregs.
(PDF)
