# Correction: FOXP3+ Regulatory T Cells in Hepatic Fibrosis and Splenomegaly Caused by *Schistosoma japonicum*: The Spleen May Be a Major Source of Tregs in Subjects with Splenomegaly

**DOI:** 10.1371/journal.pntd.0004454

**Published:** 2016-02-05

**Authors:** Audrey Romano, Xunya Hou, Mathieu Sertorio, Hélia Dessein, Sandrine Cabantous, Pablo Oliveira, Jun Li, Sandrine Oyegue, Violaine Arnaud, Xinsong Luo, Martine Daujat-Chavanieu, Odette Mariani, Xavier Sastre, Anne-Marie Dombey, Hongbin He, Yuesheng Li, Alain Dessein

The eleventh author’s name is spelled incorrectly. The correct spelling is: Martine Daujat-Chavanieu.
