# Supplementary material for: FOXP3+ Regulatory T Cells in Hepatic Fibrosis and Splenomegaly Caused by Schistosoma japonicum: The Spleen May Be a Major Source of Tregs in Subjects with Splenomegaly
Source: PLoS Negl Trop Dis. 2016 Jan 5;10(1):e0004306. doi: 10.1371/journal.pntd.0004306 (PMC4701139; doi:10.1371/journal.pntd.0004306)
Supplement: S1 Fig — A) Representative dotplos showing isotypes control antibodies staining for FOXP3 and CD45RA B) Representative dotplots of the proportions of CD25hi, CCR7+, CXCR3+, CCR5+ and CTLA-4+ cells among naïve Tregs and eTregs. (PDF) [file pntd.0004306.s001.pdf]

Fig. 1S

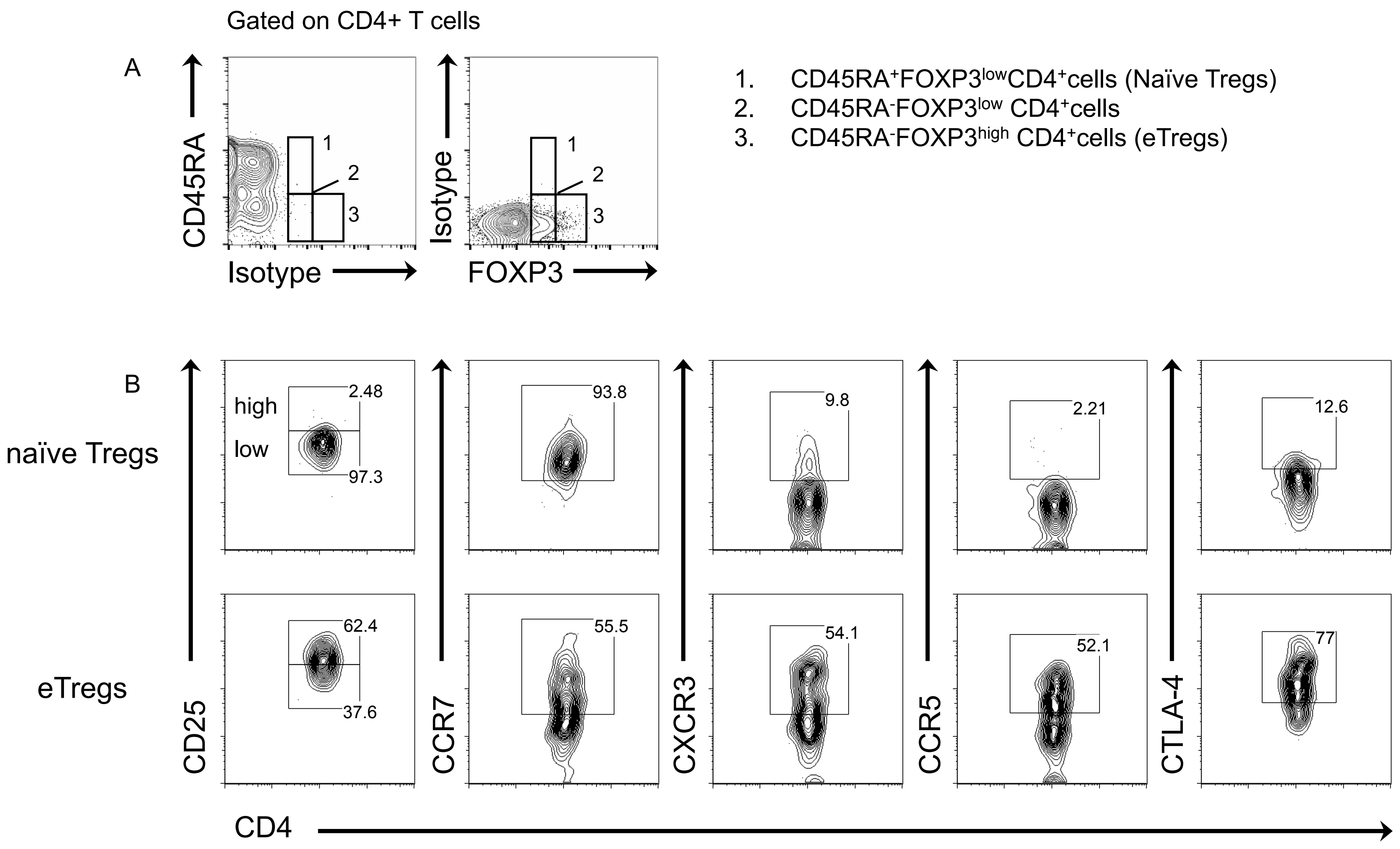

1. CD45RA$^+$FOXP3^{low}CD4$^+$cells (Naïve Tregs)
2. CD45RA$^-$FOXP3^{low} CD4$^+$cells
3. CD45RA$^-$FOXP3^{high} CD4$^+$cells (eTregs)
